# Supplementary material for: Resonance tracking in a micromechanical device using phononic frequency combs
Source: Sci Rep. 2019 Jul 1;9:9452. doi: 10.1038/s41598-019-46003-3 (PMC6602941; doi:10.1038/s41598-019-46003-3)
Supplement: Supplementary file 1 — Supplementary Information [file 41598_2019_46003_MOESM1_ESM.pdf]

Supplementary Information

Resonance tracking in a micromechanical device using phononic frequency combs

Adarsh Ganesan[1] and Ashwin Seshia[1]

[1.] Nanoscience Centre, University of Cambridge, Cambridge, UK

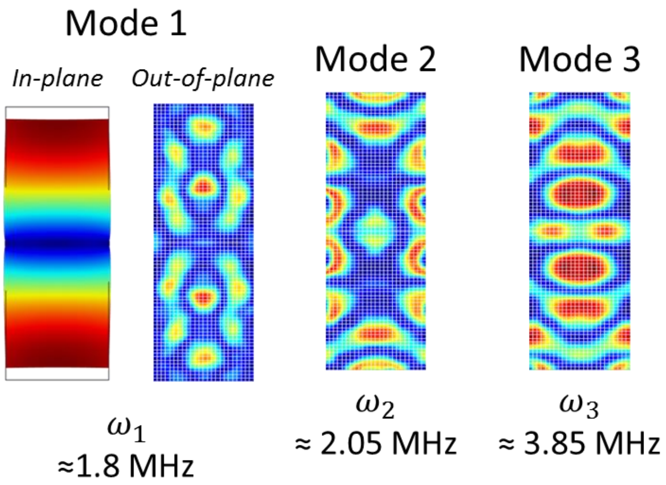

Figure S1: Modes 1-3 of the free-free beam micromechanical resonator.
